# *TOMM40 *rs2075650 polymorphism shows no association with neovascular age-related macular degeneration or polypoidal choroidal vasculopathy in a Chinese population

**Published:** 2013-09-27

**Authors:** Jing Guo, Haiping Li, Chunfang Zhang, Yaoyao Sun, Xun Deng, YuJing Bai, Shanshan Li, Min Zhao, Heng Miao, Wenzhen Yu, Bin Wang, Lvzhen Huang, Xiaoxin Li

**Affiliations:** 1 Department of Ophthalmology, Peking University People’s Hospital, Beijing, China; 2 Key Laboratory of Vision Loss and Restoration, Ministry of Education, Beijing, China; 3 Peking University Eye Center, Peking University Third Hospital, Beijing, China; 4 Department of Clinical Epidemiology, Peking University People’s Hospital, Beijing, China

## Abstract

**Purpose:**

Age-related macular degeneration (AMD) and Alzheimer disease (AD) are age-related neurodegenerative diseases that share similar environmental risk factors, cellular pathologies, and genetic backgrounds. Recently, the rs2075650 single nucleotide polymorphism in the translocase of outer mitochondrial membrane 40 homolog (*TOMM40*) gene was identified as a risk factor for AMD and Alzheimer disease. We aimed to examine the associations between the *TOMM40* rs2075650 polymorphism and neovascular age-related macular degeneration (nAMD) and polypoidal choroidal vasculopathy (PCV) in a Chinese population.

**Methods:**

The study consisted of 900 subjects, including 300 controls, 300 cases with nAMD, and 300 cases with PCV. Genomic DNA was extracted from venous blood leukocytes. The allelic variant of rs2075650 was determined with matrix-assisted laser desorption/ionization time-of-flight mass spectrometry. Differences in the observed genotypic distributions between the case and control groups were tested using chi-square tests, with age and gender adjusted using logistic regression analysis.

**Results:**

The *TOMM40* rs2075650 polymorphism was not statistically significantly associated with the nAMD or PCV phenotype (p>0.05). The difference remained insignificant after correction for age and gender differences based on the logistic regression models (p>0.05).

**Conclusions:**

Our data provide no evidence to support an association of rs2075650 in *TOMM40* with nAMD or PCV, suggesting that this gene is unlikely to be a major AMD and PCV susceptibility gene locus in the Chinese population.

## Introduction

Age-related macular degeneration (AMD), which causes irreversible central vision loss, is the most common cause of legal blindness in the elderly in developed countries [1]. AMD is characterized as a chronic and progressive degeneration of photoreceptors, the underlying retinal pigment epithelium, Bruch’s membrane, and potentially, the choriocapillaris in the macula [2]. Choroidal neovascularization (CNV), the hallmark of wet, or neovascular, AMD, is the main cause of severe vision loss due to AMD [3]. Polypoidal choroidal vasculopathy (PCV) is associated with a decrease in vision in the elderly Asian population, and is characterized by a network of vessels with two distinct components: a complex of branching vessels and multiple, terminal, reddish-orange polypoidal lesions [4-6]. PCV has been described as a distinct clinical entity from AMD and the other diseases associated with subretinal neovascularization [7]. Nevertheless, whether PCV represents a subtype of neovascular AMD remains controversial [8]. Evidence suggests that AMD and PCV, despite their different phenotypic manifestations, may share common genetic risk factors [9-13].

The translocase of the outer mitochondrial membrane 40 (*TOMM40*) gene has been proposed as a genetic risk factor in Alzheimer disease (AD). Recently, several single nucleotide polymorphisms (SNPs) in the *TOMM40* gene showed a highly significant association with AD in published genome-wide association studies (GWASs) [14-18]. A *TOMM40* risk allele, rs2075650, showed compelling evidence of association with risk in AD [15,18,19]. *TOMM40* encodes Tom40, a channel-forming subunit of the multisubunit translocase of the outer mitochondrial membrane (TOM complex), which plays a role in cytoplasmic peptide and protein transport into the mitochondria [20]. Additionally, the amyloid-β peptide (Aβ) was reported to be transported into mitochondria via the TOM machinery and to subsequently accumulate in the mitochondrial cristae, which may result in mitochondrial dysfunction and promote neurotoxicity in AD [20]. Taken together, there is genetic and physiologic evidence for the involvement of *TOMM40* in AD risk and pathogenesis. AMD and AD are age-related neurodegenerative diseases that share similar environmental risk factors, cellular pathologies, and genetic backgrounds [21-25]. Furthermore, Holliday et al. demonstrated that rs2075650 in the *TOMM40* gene was associated with early AMD in subjects with European and Singaporean ancestry [26]. However, thus far, no AMD or PCV association studies of rs2075650 have been performed in the Chinese population; it is imperative to investigate further. Therefore, we analyzed rs2075650 in the *TOMM40* gene to determine any potential influence on AMD or PCV risk in our Chinese population.

## Methods

### Subjects

Nine hundred unrelated Chinese subjects were studied in this case-control cohort. Three hundred patients had nAMD, and three hundred patients had polypoidal choroidal vasculopathy (PCV). Three hundred individuals without age-related maculopathy (ARM) were studied as controls. The genders and ages of the controls and cases are given in Table 1. The study participants were recruited at the Department of Ophthalmology in the Peking University People’s Hospital, and the study was approved by the Ethical Committee of Peking University People’s Hospital. An informed consent process was established following the guidelines of the Helsinki Declaration, and consent forms were signed by all subjects. All subjects received a comprehensive ophthalmic examination, including visual acuity measurements, slit-lamp biomicroscopy, and dilated fundus examination performed by a retinal specialist. All cases with nAMD and PCV underwent fluorescein angiography, optic coherence tomography (OCT), and indocyanine green angiograms with HRA2 (Heidelberg Engineering, Heidelberg, Germany). The diagnosis of nAMD or ARM was defined by the International Classification System for ARM [27]. The diagnosis of PCV was based on indocyanine green angiography results that showed a branching vascular network terminating in aneurysmal enlargements, which typify polypoidal lesions. Exclusion criteria included any eye with any other macular abnormalities, such as pathologic myopia, idiopathic choroidal neovascularization (CNV), presumed ocular histoplasmosis, angioid streaks, and any other secondary CNV. Normal controls were defined as having no clinical evidence of AMD or PCV in either eye or any other eye diseases, excluding mild age-related cataracts. Subjects with severe cataracts were excluded from the study.

**Table 1:** Demographic Distribution of the study subjects

**Parameters**	**nAMD (n=300)**	**PCV (n=300)**	**Controls (n=300)**
**Females, n (%)**	111(37.0%)	112(37.3%)	158(52.7%)
**Males, n (%)**	189(63.0%)	188(62.7%)	142(47.3%)
Age* range (Years)	50–90	42–85	45–95
Mean age±SD** (Years)	69.4±8.9	66.8±9.7	65.1±9.5

* Age of presentation. ** SD, standard deviation.

### Genetic analysis

Blood samples were collected from all participants and stored at −80 °C before DNA was extracted. Genomic DNA was extracted from venous blood leukocytes using a genomic extraction kit (Beijing eBios Biotechnology, Beijing, China), and genotyping was performed with matrix-assisted laser desorption/ionization time-of-flight mass spectrometry, as previously described [28]. Briefly, approximately 30 ng of genomic DNA was used to genotype each sample. The primer sequences were 5′-ACG TTG GAT GAG CTC TGG AGA AGA GAA ACG-3′ and 5′-ACG TTG GAT GTG GTA GGG AAG GAA GAG ATG-3′. The DNA samples were amplified, and the PCR products were used for locus-specific single-base extension reactions. The resulting products were desalted and transferred to a 384 SpectroCHIP array (Sequenom, San Diego, CA). Allele detection was performed using matrix-assisted laser desorption/ionization time-of-flight mass spectrometry. The mass spectrograms were analyzed using MassARRAY Typer software version 4.0 (Sequenom, San Diego, CA).

### Statistical analysis

The data were analyzed using SPSS (version 16.0; SPSS Science, Chicago, IL). All identified polymorphisms were assessed for Hardy–Weinberg equilibrium using chi-square tests. Single-marker association analyses were performed using chi-square tests or Fisher’s exact tests under various genetic models. Logistic regression models were used to calculate the odds ratio (OR) and 95% confidence interval of nAMD or PCV, comparing the case groups to the control group. Age- and gender-adjusted p values and ORs were also calculated. The statistical power was also calculated. Values of p<0.05 were considered statistically significant.

## Results

A total of 900 subjects participated in the study, including 300 control subjects (mean age±standard deviation [SD], 65.1±9.5 years; ratio of women to men, 52.7:47.3), 300 cases with nAMD (mean age±SD, 69.4±8.9 years; ratio of women to men, 37.0:63.0) in one or both eyes, and 300 cases with PCV (mean age±SD, 66.8±9.7 years; ratio of women to men, 37.3:62.7) in at least one eye. The general characteristics of the study subjects are in Table 1.

The *TOMM40* rs2075650 allele and genotype counts and results of single-SNP association analysis are in Table 2. The SNP did not show a significant association with either nAMD or PCV in the genotype, allele, or any of the genetic models (all p>0.05; Table 2). Even after correction for age, gender, different genotypes, and various genetic models based on a logistic regression model, the associations remained insignificant (all p>0.05; Table 3). The statistical powers to detect the association between the SNP and nAMD or PCV ranged from 0.05 to 0.10, assuming conventional levels of statistical significance (powers not available in the recessive model; Table 2). The frequencies of all genotypes were under Hardy–Weinberg equilibrium in the controls and the cases with nAMD or PCV (all p>0.05, Appendix 1).

**Table 2:** The *TOMM40* rs2075650 genotype and allele frequency distribution and the results of association tests.

**Frequency Distribution**					**nAMD versus Control**			**PCV versus Control**		
**Parameters**		**nAMD**	**PCV**	**Control**	**P^†^**	**OR(95%CI)^‡^**	**Power**^§^	**P^†^**	**OR(95%CI)^‡^**	**Power**^§^
**Genotype**	**AA**	251(83.7)	244(81.3)	250(83.3)	1	-		845	-	
	**GA**	47(15.7)	54(18.0)	48(16.0)						
	**GG**	2(0.7)	2(0.7)	2(0.7)						
**Allele**	**A**	549(91.5)	542(90.3)	548(91.3)	0.918	0.979(0.654,1.466)	0.051	0.548	1.128(0.761,1.670)	0.080
	**G**	51(8.5)	58(9.7)	52(8.7)						
Dominant*	**AA**	251(83.7)	244(81.3)	250(83.3)	0.912	0.976(0.634,1.502)	0.052	0.521	1.148(0.754,1.747)	0.098
	**GA+GG**	49(16.3)	56(18.7)	50(16.7)						
Recessive**	**AA+GA**	298(99.3)	298(99.3)	298(99.3)	1	1.000(0.140, 7.146)	-	1	1.000(0.140, 7.146)	-
	**GG**	2(0.7)	2(0.7)	2(0.7)						

* The dominant model compared a combination of heterozygotes and rare homozygotes to the common homozygotes. ** The recessive model compared the rare homozygotes to a combination of common homozygotes and heterozygotes. ^†^ p<0.05 was considered significant. ^‡^ OR, odds ratio; CI, confidence interval. ^§^ Statistical power for detecting the association.

**Table 3:** The adjusted p values and odds ratios for age, gender, different genotypes and other various genetic models in each disease.

**Parameters**		**nAMD versus Control**		**PCV versus Control**	
		**P^†^**	**OR(95%CI)^‡^**	**P^†^**	**OR(95%CI)^‡^**
**Genotype**	**AA**	-	1.00	-	1.00
	**GA**	0.663	0.903(0.571,1.428)	0.513	1.156(0.749,1.783)
	**GG**	0.916	1.118(0.139,8.992)	0.884	1.160(0.157,8.583)
**Genetic model**	Dominant*	0.663	0.903(0.571,1.428)	0.513	1.156(0.749,1.783)
	Recessive**	0.843	1.238(0.149,10.308)	0.997	1.004(0.132,7.655)

* The dominant model compared a combination of heterozygotes and rare homozygotes to the common homozygotes. ** The recessive model compared the rare homozygotes to a combination of common homozygotes and heterozygotes. ^†^ p<0.05 was considered significant. ^‡^ OR, odds ratio; CI, confidence interval.

## Discussion

To our knowledge, this is the first investigation of an association between the well-known *TOMM40* rs2075650 polymorphism and nAMD and PCV risk in a large Chinese sample. The results of this study showed that rs2075650 was not associated with increased risk for nAMD or PCV in the Chinese population.

Disease progression is characterized pathologically by the progressive appearance of proinflammatory, highly insoluble, amyloid-beta peptide (Aβ)-enriched pathological lesions known as neocortical senile plaques in the brain parenchyma and retinal drusen in the macular region in AD and AMD, respectively. Aging, hypercholesterolemia, hypertension, obesity, arteriosclerosis, and smoking are risk factors for AMD and AD [22-24]. Nevertheless, the genetic risk factors in AMD and AD seem to have different origins, although polymorphisms in the apolipoprotein E and complement factor H genes have shown effects on the risks for AMD and AD [21-25,29]. Our study provided further support to the hypothesis that although the rs2075650 polymorphism is associated with AD [15,18,19], this polymorphism is not involved in nAMD.

It was previously observed that the rs2075650 polymorphism was associated with AMD. A GWAS meta-analysis of early AMD was conducted, which included approximately 4,000 early AMD cases and 20,000 strictly defined controls with no drusen or with hard drusen only. This meta-analysis amalgamated several large population-based cohorts with GWAS data, including European and Asian (Singaporean) ancestry. The *TOMM40* rs2075650 polymorphism was detected as a confirmed and plausible candidate SNP, demonstrating a possible association with early AMD (p=1.1E-06) [26]. However, the frequency of the A allele was reported to be 86.0% in the European and the Singaporean populations in the previous meta-analysis [26]. In the current study, the frequency of the A allele was 91.1% in all subjects. Whether this ethnic difference influenced the power of our study to replicate the previously reported findings remains to be investigated. In fact, regarding the allele frequency data from the HapMap, the rs2075650 SNP in *TOMM40* shows a greater major allele frequency in Chinese than in Caucasians. Another possible explanation for the differences between our findings and the previous study may include differences in the stages and phenotypes of AMD. Early AMD was defined as the presence of soft drusen (>63 µm) alone, retinal pigment epithelium depigmentation alone, or a combination of soft drusen with increased retinal pigment and/or depigmentation in the absence of late AMD in the previous GWAS meta-analysis [26]. NAMD is characterized by the development of CNV [3]. The differences in some genetic variants and their relative contributions to the development of either exudative or atrophic AMD or the types of drusen have been noted [30-33]. Numerous studies have reported that the distinguishing features of Asian AMD include male predominance, unilateral presentation, a comparatively low incidence of soft drusen, and a greater prevalence of neovascular AMD and PCV [34-36]. In our study, male prevalence in the nAMD and PCV groups was 63.0% and 62.7%, respectively, and was significantly higher than in the controls (p<0.001, Appendix 2). Because of significance in gender distribution, the following genotyping analyses were adjusted by gender using logistic regression analysis. Nevertheless, even after correction for gender, the associations remained insignificant.

Some investigators have hypothesized that true disease-associated SNPs would be more likely to have nearby disease-associated SNPs (due to linkage disequilibrium). They proposed the idea that features of individual SNPs associated with disease and clustering of nearby significant SNPs would be useful in selecting loci from genetic association studies for replication [37,38]. In recent years, the apolipoprotein E SNPs rs429358 and rs7412 have been reported to associate with AMD [39-41]. Based on the theory mentioned above, the *TOMM40* SNP rs2075650, which is in linkage disequilibrium with them, might be a susceptibility locus [42]. However, our study failed to confirm this hypothesis.

To the best of our knowledge, no report has been published on the possible association between the *TOMM40* rs2075650 polymorphism and PCV. Our data provide no evidence to support an association between them.

Another possible explanation is the inadequate statistical power to detect a potential association between rs2075650 and nAMD or PCV. Based on the original study reported by Holliday et al. (OR=1.23) [26] and the allele frequency in the current controls, the required sample size is 5,036 (n_case_=n_control_=5036) with a power of 0.800. However, one issue in our analysis merited careful consideration. The odds ratio for the nAMD and control group was close to unity (0.979), which indicated only a weak association. We therefore did not further enlarge the sample size.

Alternatively, the fact that genetic variants associated with a particular disease in this population may not necessarily be associated in other populations must be considered. Moreover, the gene-disease association of rs2075650 in populations from East Asia could be weak or absent when compared with Caucasian populations.

In summary, we found no evidence to support a significant association of the *TOMM40* rs2075650 polymorphism with nAMD or PCV in this study, suggesting that this polymorphism is unlikely to be a major susceptibility gene locus for these diseases in the Chinese population. Whether there is a weak association between the *TOMM40* rs2075650 polymorphism and nAMD or PCV in Chinese must be confirmed with future studies with much larger sample sizes.

## Appendix 1. Hardy–Weinberg equilibrium values for all the genotypes.

* HWE, Hardy–Weinberg equilibrium. To access the data, click or select the words “Appendix 1.”

## Appendix 2. The p values and odds ratios for gender in each disease.

^†^ p<0.05 was considered significant. ^‡^ OR, odds ratio; CI, confidence interval. To access the data, click or select the words “Appendix 2.”

## References

[1] Lim LS, Mitchell P, Seddon JM, Holz FG, Wong TY (2012). Age-related macular degeneration.. Lancet.

[2] Jager RD, Mieler WF, Miller JW (2008). Age-related macular degeneration.. N Engl J Med.

[3] Sayen A, Hubert I, Berrod JP (2011). Age related macular degeneration.. Rev Prat.

[4] Yannuzzi LA, Ciardella A, Spaide RF, Rabb M, Freund KB, Orlock DA (1997). The expanding clinical spectrum of idiopathic polypoidal choroidal vasculopathy.. Arch Ophthaloml.

[5] Imamura Y, Engelbert M, Iida T, Freund KB, Yannuzzi LA (2010). Polypoidal choroidal vasculopathy: a review.. Surv Ophthalmol.

[6] Spaide RF, Yannuzzi LA, Slakter JS, Sorenson J, Orlach DA (1995). Indocyanine green videoangiography of idiopathic polypoidal choroidal vasculopathy.. Retina.

[7] Ciardella AP, Donsoff IM, Huang SJ, Costa DL, Yannuzzi LA (2004). Polypoidal choroidal vasculopathy.. Surv Ophthalmol.

[8] Terasaki H, Miyake Y, Suzuki T, Nakamura M, Nagasaka T (2002). Polypoidal choroidal vasculopathy treated with macular translocation: clinical pathological correlation.. Br J Ophthalmol.

[9] Kondo N, Honda S, Ishibashi K, Tsukahara Y, Negi A (2007). LOC387715/HTRA1 variants in polypoidal choroidal vasculopathy and age-related macular degeneration in a Japanese population.. Am J Ophthalmol.

[10] Gotoh N, Yamada R, Nakanishi H, Saito M, Iida T, Matsuda F, Yoshimura N (2008). Correlation between CFH Y402H and HTRA1 rs11200638 genotype to typical exudative age-related macular degeneration and polypoidal choroidal vasculopathy phenotype in the Japanese population.. Clin Experiment Ophthalmol.

[11] Bessho H, Kondo N, Honda S, Kuno S, Negi A (2009). Coding variant Met72Thr in the PEDF gene and risk of neovascular age-related macular degeneration and polypoidal choroidal vasculopathy.. Mol Vis.

[12] Lima LH, Schubert C, Ferrara DC, Merriam JE, Imamura Y, Freund KB, Spaide RF, Yannuzzi LA, Allikmets R (2010). Three major loci involved in age-related macular degeneration are also associated with polypoidal choroidal vasculopathy.. Ophthalmology.

[13] Cheng Y, Huang L, Li X, Zhou P, Zeng W, Zhang C (2013). Genetic and Functional Dissection of A RMS2 in Age-Related Macular Degeneration and Polypoidal Choroidal Vasculopathy.. PLoS ONE.

[14] Harold D, Abraham R, Hollingworth P, Sims R, Gerrish A, Hamshere ML, Pahwa JS, Moskvina V, Dowzell K, Williams A, Jones N, Thomas C, Stretton A, Morgan AR, Lovestone S, Powell J, Proitsi P, Lupton MK, Brayne C, Rubinsztein DC, Gill M, Lawlor B, Lynch A, Morgan K, Brown KS, Passmore PA, Craig D, McGuinness B, Todd S, Holmes C, Mann D, Smith AD, Love S, Kehoe PG, Hardy J, Mead S, Fox N, Rossor M, Collinge J, Maier W, Jessen F, Schürmann B, van den Bussche H, Heuser I, Kornhuber J, Wiltfang J, Dichgans M, Frölich L, Hampel H, Hüll M, Rujescu D, Goate AM, Kauwe JS, Cruchaga C, Nowotny P, Morris JC, Mayo K, Sleegers K, Bettens K, Engelborghs S, De Deyn PP, Van Broeckhoven C, Livingston G, Bass NJ, Gurling H, McQuillin A, Gwilliam R, Deloukas P, Al-Chalabi A, Shaw CE, Tsolaki M, Singleton AB, Guerreiro R, Mühleisen TW, Nöthen MM, Moebus S, Jöckel KH, Klopp N, Wichmann HE, Carrasquillo MM, Pankratz VS, Younkin SG, Holmans PA, O'Donovan M, Owen MJ, Williams J (2009). Genome-wide association study identifies variants at CLU and PICALM associated with Alzheimer’s disease.. Nat Genet.

[15] Potkin SG, Guffanti G, Lakatos A, Turner JA, Kruggel F, Fallon JH, Saykin AJ, Orro A, Lupoli S, Salvi E, Weiner M, Macciardi F (2009). Alzheimer's Disease Neuroimaging Initiative. Hippocampal atrophy as a quantitative trait in a genome-wide association study identifying novel susceptibility genes for Alzheimer's disease.. PLoS ONE.

[16] Shen L, Kim S, Risacher SL, Nho K, Swaminathan S, West JD, Foroud T, Pankratz N, Moore JH, Sloan CD, Huentelman MJ, Craig DW, Dechairo BM, Potkin SG, Jack CR, Weiner MW, Saykin AJ, Alzheimer's Disease Neuroimaging Initiative (2010). Whole genome association study of brain-wide imaging phenotypes for identifying quantitative trait loci in MCI and AD: A study of the ADNI cohort.. Neuroimage.

[17] Kim S, Swaminathan S, Shen L, Risacher SL, Nho K, Foroud T, Shaw LM, Trojanowski JQ, Potkin SG, Huentelman MJ, Craig DW, DeChairo BM, Aisen PS, Petersen RC, Weiner MW, Saykin AJ, Alzheimer's Disease Neuroimaging Initiative (2011). Genome-wide association study of CSF biomarkers Abeta1–42, t-tau, and p-tau181p in the ADNI cohort.. Neurology.

[18] Ma XY, Yu JT, Wang W, Wang HF, Liu QY, Zhang W, Tan L (2013). Association of TOMM40 Polymorphisms with Late-Onset Alzheimer's Disease in a Northern Han Chinese Population.. Neuromolecular Med.

[19] Naj AC, Beecham GW, Martin ER, Gallins PJ, Powell EH, Konidari I, Whitehead PL, Cai G, Haroutunian V, Scott WK, Vance JM, Slifer MA, Gwirtsman HE, Gilbert JR, Haines JL, Buxbaum JD, Pericak-Vance MA (2010). Dementia revealed: novel chromosome 6 locus for late-onset Alzheimer disease provides genetic evidence for folate-pathway abnormalities.. PLoS Genet.

[20] Humphries AD, Streimann IC, Stojanovski D, Johnston AJ, Yano M, Hoogenraad NJ, Ryan MT (2005). Dissection of the mitochondrial import and assembly pathway for human Tom40.. J Biochem.

[21] Malek G, Johnson LV, Mace BE, Saloupis P, Schmechel DE, Rickman DW, Toth CA, Sullivan PM, Bowes Rickman C (2005). Apolipoprotein E allele-dependent pathogenesis: a model for age-related retinal degeneration.. Proc Natl Acad Sci USA.

[22] Ohno-Matsui K (2011). Parallel findings in age-related macular degeneration and Alzheimer's disease.. Prog Retin Eye Res.

[23] Lukiw WJ, Surjyadipta B, Dua P, Alexandrov PN (2012). Common micro RNAs (miRNAs) target complement factor H (CFH) regulation in Alzheimer's disease (AD) and in age-related macular degeneration (AMD).. Int J Biochem Mol Biol.

[24] Kaarniranta K, Salminen A, Haapasalo A, Soininen H, Hiltunen M (2011). Age-related macular degeneration (AMD): Alzheimer's disease in the eye?. J Alzheimers Dis.

[25] Zetterberg M, Landgren S, Andersson ME, Palmér MS, Gustafson DR, Skoog I, Minthon L, Thelle DS, Wallin A, Bogdanovic N, Andreasen N, Blennow K, Zetterberg H (2008). Association of complement factor H Y402H gene polymorphism with Alzheimer's disease.. Am J Med Genet B Neuropsychiatr Genet.

[26] Holliday EG, Smith AV, Cornes BK, Buitendijk GH, Jensen RA, Sim X, Aspelund T, Aung T, Baird PN, Boerwinkle E, Cheng CY, van Duijn CM, Eiriksdottir G, Gudnason V, Harris T, Hewitt AW, Inouye M, Jonasson F, Klein BE, Launer L, Li X, Liew G, Lumley T, McElduff P, McKnight B, Mitchell P, Psaty BM, Rochtchina E, Rotter JI, Scott RJ, Tay W, Taylor K, Teo YY, Uitterlinden AG, Viswanathan A, Xie S (2013). Wellcome Trust Case Control Consortium 2, Vingerling JR, Klaver CC, Tai ES, Siscovick D, Klein R, Cotch MF, Wong TY, Attia J, Wang JJ. Insights into the Genetic Architecture of Early Stage Age-Related Macular Degeneration: A Genome-Wide Association Study Meta-Analysis.. PLoS ONE.

[27] Bird AC, Bressler NM, Bressler SB, Chisholm IH, Coscas G, Davis MD, de Jong PT, Klaver CC, Klein BE, Klein R, Mitchell P, Sarks JP, Sarks SH, Soubrane G, Taylor HR, Vingerling JR, The International ARM Epidemiological study group (1995). An international classification and grading system for age-related maculopathy and age-related macular degeneration.. Surv Ophthalmol.

[28] Schaeffeler E, Zanger UM, Eichelbaum M, Asante-Poku S, Shin JG, Schwab M (2008). Highly multiplexed genotyping of thiopurine s-methyltransferase variants using MALD-TOF mass spectrometry: reliable genotyping in different ethnic groups.. Clin Chem.

[29] Proitsi P, Lupton MK, Dudbridge F, Tsolaki M, Hamilton G, Daniilidou M, Pritchard M, Lord K, Martin BM, Johnson J, Craig D, Todd S, McGuinness B, Hollingworth P, Harold D, Kloszewska I, Soininen H, Mecocci P, Velas B, Gill M, Lawlor B, Rubinsztein DC, Brayne C, Passmore PA, Williams J, Lovestone S, Powell JF (2012). Alzheimer's disease and age-related macular degeneration have different genetic models for complement gene variation.. Neurobiol Aging.

[30] Farwick A, Wellmann J, Stoll M, Pauleikhoff D, Hense HW (2010). Susceptibility genes and progression in age-related maculopathy: a study of single eyes.. Invest Ophthalmol Vis Sci.

[31] Sobrin L, Reynolds R, Yu Y, Fagerness J, Leveziel N, Bernstein PS, Souied EH, Daly MJ, Seddon JM (2011). ARMS2/HTRA1 locus can confer differential susceptibility to the advanced subtypes of age-related macular degeneration.. Am J Ophthalmol.

[32] Boon CJ, Klevering BJ, Hoyng CB, Zonneveld-Vrieling MN, Nabuurs SB, Blokland E, Cremers FP, den Hollander AI (2008). Basal laminar drusen caused by compound heterozygous variants in the CFH gene.. Am J Hum Genet.

[33] Seddon JM, Reynolds R, Rosner B (2009). Peripheral retinal drusen and reticular pigment: association with CFHY402H and CFHrs1410996 genotypes in family and twin studies.. Invest Ophthalmol Vis Sci.

[34] Chang TS, Hay D, Courtright P (1999). Age-related macular degeneration in Chinese-Canadians.. Can J Ophthalmol.

[35] Bird AC (2003). The Bowman lecture. Towards an understanding of age-related macular disease.. Eye (Lond).

[36] Hou J, Tao Y, Li XX, Zhao MW (2011). Clinical characteristics of polypoidal choroidal vasculopathy in Chinese patients.. Graefes Arch Clin Exp Ophthalmol.

[37] Teo YY (2008). Common statistical issues in genome-wide association studies: a review on power, data quality control, genotype calling and population structure.. Curr Opin Lipidol.

[38] Edwards AO, Fridley BL, James KM, Sharma AK, Cunningham JM, Tosakulwong N (2008). Evaluation of clustering and genotype distribution for replication in genome wide association studies: the age-related eye disease study.. PLoS ONE.

[39] Klaver CC, Kliffen M, van Duijn CM, Hofman A, Cruts M, Grobbee DE, van Broeckhoven C, de Jong PT (1998). Genetic association of apolipoprotein E with age-related macular degeneration.. Am J Hum Genet.

[40] Zareparsi S, Reddick AC, Branham KE, Moore KB, Jessup L, Thoms S, Smith-Wheelock M, Yashar BM, Swaroop A (2004). Association of apolipoprotein E alleles with susceptibility to age-related macular degeneration in a large cohort from a single center.. Invest Ophthalmol Vis Sci.

[41] McKay GJ, Patterson CC, Chakravarthy U, Dasari S, Klaver CC, Vingerling JR, Ho L, de Jong PT, Fletcher AE, Young IS, Seland JH, Rahu M, Soubrane G, Tomazzoli L, Topouzis F, Vioque J, Hingorani AD, Sofat R, Dean M, Sawitzke J, Seddon JM, Peter I, Webster AR, Moore AT, Yates JR, Cipriani V, Fritsche LG, Weber BH, Keilhauer CN, Lotery AJ, Ennis S, Klein ML, Francis PJ, Stambolian D, Orlin A, Gorin MB, Weeks DE, Kuo CL, Swaroop A, Othman M, Kanda A, Chen W, Abecasis GR, Wright AF, Hayward C, Baird PN, Guymer RH, Attia J, Thakkinstian A, Silvestri G (2011). Evidence of association of APOE with age-related macular degeneration: a pooled analysis of 15 studies.. Hum Mutat.

[42] Deelen J, Beekman M, Uh HW, Helmer Q, Kuningas M, Christiansen L, Kremer D, van der Breggen R, Suchiman HE, Lakenberg N, van den Akker EB, Passtoors WM, Tiemeier H, van Heemst D, de Craen AJ, Rivadeneira F, de Geus EJ, Perola M, van der Ouderaa FJ, Gunn DA, Boomsma DI, Uitterlinden AG, Christensen K, van Duijn CM, Heijmans BT, Houwing-Duistermaat JJ, Westendorp RG, Slagboom PE (2011). Genome-wide association study identifies a single major locus contributing to survival into old age; the APOE locus revisited.. Aging Cell.

